# Review: Effects of anti‐CD38 monoclonal antibodies on red blood cell transfusion and interventions

**DOI:** 10.1002/jcla.23832

**Published:** 2021-11-09

**Authors:** Jia Song, Rong Fu

**Affiliations:** ^1^ Department of Hematology General Hospital Tianjin Medical University Tianjin China

**Keywords:** CD38, multiple myeloma, transfusion

## Abstract

**Background:**

Highly expressed in almost all myeloma cells, CD38 is an attractive treatment target.

**Aim:**

Anti‐CD38 monoclonal antibodies have been approved for first‐line treatment in non‐transplantable multiple myeloma (MM) patients.

**Materials and methods:**

However, it has been found in clinical use that anti‐CD38 monoclonal antibodies bind to CD38 on red blood cells (RBCs) and cause panagglutination in indirect antiglobulin test (IAT), resulting in false positives of IAT (Transfusion, 55, 2015 and 1545; Transfusion, 55, 2015 and 1555).

**Result:**

Thereby, interfering with blood bank testing and leading to the delay of further diagnosis and treatment.

**Conclusion:**

With more and more patients receiving anti‐CD38 treatment, it is of great importance to recognize this problem and optimize relevant diagnosis and treatment procedures to prevent RBC transfusion delays and reduce laboratory costs.

## Graphical abstract

Anti‐CD38 monoclonal antibodies for multiple myeloma (MM). However, it bind to CD38 on RBCs and cause pan agglutination, resulting in false positives of IAT. It is of great importance to recognize this problem and optimize relevant diagnosis and treatment procedures to prevent RBC transfusion delays and reduce laboratory costs. This article will review the mechanisms and the interventions of the impact from anti‐CD38 monoclonal antibodies on red blood cell transfusion.

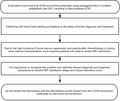

## 1 INTRODUCTION

Highly expressed in almost all myeloma cells, CD38 is an attractive treatment target. Anti‐CD38 monoclonal antibodies have been approved for first‐line treatment in non‐transplantable multiple myeloma (MM) patients. In the future, they are likely to be approved for more indications. However, it has been found in clinical use that anti‐CD38 monoclonal antibodies bind to CD38 on red blood cells (RBCs) and cause pan agglutination in indirect antiglobulin test (IAT), resulting in false positives of IAT^1^, ^2^ and, thereby, interfering with blood bank testing and leading to the delay of further diagnosis and treatment. Due to the high incidence of bone marrow suppression and anemia after chemotherapy or during bone marrow transplantation, most myeloma patients will need to receive RBC transfusion. With more and more patients receiving anti‐CD38 treatment, it is of great importance to recognize this problem and optimize relevant diagnosis and treatment procedures to prevent RBC transfusion delays and reduce laboratory costs. In this article, we will review the mechanisms and the interventions of the impact from anti‐CD38 monoclonal antibodies on red blood cell transfusion.

## 2 MECHANISM

In multiple domestic and foreign studies, it was found that, following treatment with anti‐CD38 monoclonal antibodies for 2–6 months, IAT was falsely positive in patients, resulting in positive antibody screens and incompatible crossmatches. This phenomenon occurs following the administration of daratumumab and isatuximab, suggesting that it is not drug‐specific but associated with the anti‐CD38 characteristics of the drugs.

CD38 is a transmembrane glycoprotein closely relevant to physiological functions including calcium regulation, signal transduction, and cell adhesion.^3^ Considering the extensive and high‐level expression of CD38 in myeloma cells, monoclonal antibodies targeting CD38 have been developed, which have proven significantly active in the treatment of MM. Anti‐CD38 monoclonal antibodies act by multiple mechanisms such as triggering complement‐dependent cytotoxicity, antibody‐dependent cell‐mediated cytotoxicity, or antibody‐dependent cell phagocytosis, inducing apoptosis or influencing the immune regulatory functions of regulatory T cells and cytotoxic T cells.^3^ In addition to myeloma cells, CD38 also expresses on the RBC membrane. This is why anti‐CD38 monoclonal antibodies induce agglutination in IAT.

Indirect antiglobulin test uses normal RBCs to detect incomplete antibodies in serum. Following the administration of an anti‐CD38 monoclonal antibody, the anti‐CD38 monoclonal antibody in serum will play the role of incomplete antibodies. The anti‐CD38 monoclonal antibody will sensitize RBC by specifically binding to CD38 at low expression levels on RBC surfaces. When the antiglobulin antibody is added, it will bind to the Fc fragment of the anti‐CD38 monoclonal antibody, causing weak panagglutination (usually 1+) of sensitized RBC and thus resulting in positive antibody screens and incompatible crossmatches.

The direct antiglobulin test (DAT) is usually negative in patients treated with anti‐CD38 monoclonal antibodies. This is possibly because CD38 expression on RBC surfaces is downregulated following the administration of anti‐CD38 monoclonal antibodies.^4^ In addition, following the use of anti‐CD38 monoclonal antibodies, few patients develop overt hemolysis. This is possibly associated with the low expression of CD38 on RBC surfaces.^5^ Despite the effects on the screening of irregular antibodies, the use of anti‐CD38 monoclonal antibodies usually has no impact on ABO and Rh blood typing.^6^

## 3 INTERVENTIONS

### 3.1 Processing of reagent RBCs

A study shows that daratumumab (DARA)'s interference with blood matching is effectively eliminated by RBCs inactivated with dithiothreitol (DTT).^7^ DTT denatures the CD38 antigen and prevents antibody binding by cleaving the disulfide bond on the CD38 receptor. This processing method has been verified in a multicenter study^8^ and is the most effective method currently available. However, the commonly used 0.2 mol/L DTT denatures other RBC antigens in addition to CD38. The most notably affected one is Kell, and other antigens with low immunogenicity, including antigens in Lutheran, Yt, JMH, LW, Cromer, Indian, Dombrock, and Knops systems,^9^ may also be denatured. Luckily, practically no Chinese is K antigen positive. The probability of transfusion reactions caused by DTT‐destroyed minor antigens is meager, but caution is warranted. And the risk can be eliminated by transfusing RBCs without K antigen.^1^ The latest study shows that, in microcolumn gel IAT, processing of RBCs with 0.04 mol/L DTT for 15 min can eliminate DARA interference without causing the denaturation of irregular antigens such as Kell, Lutheran, Cartwright, and JMH.^10^

Processing RBCs with proteolytic enzymes such as trypsin may also reduce the binding of DARA to CD38 on RBCs, but it is currently considered that they cannot supersede DTT. Chapuy et al^1^ found that 2% trypsin reduced the binding of Daratumumab to CD38 transformed HL‐60 cells by 40%, while the reduction could reach up to 92% with 10 mmol/L DTT. Trypsin does not degrade Kell antigen but may destroy many other clinically significant antigens, including M, N, EnaTS and low‐immunogenicity Ge2, Ge3, Ge4, Ch/Rg, and Lutheran antigens.

CD38 deficient RBCs do not bind to anti‐CD38 monoclonal antibodies and thus there will be no panagglutination in IAT. Studies indicate neonatal cord blood may be deficient of CD38 antigen. In a clinical trial, RBC antigen phenotype screening was performed using cord blood and RBC transfusion was successfully completed without transfusion reactions.^11^

### 3.2 Interference with anti‐CD38 monoclonal antibodies in serum

Complete fragmentation of Daratumumab into Fc and Fab fragments is performed by papain proteolysis. Fab fragment prevents RBC agglutination induced by FC segment by binding to CD38 on reagent RBCs.^12^ Papain degrades antigens in Duffy and MNS blood group systems and also some minor antigens including Ch/Rg, Ge2, and Ge4 (24) but will not denature Kell antigen. Therefore, it may be used in combination with DTT for antigen identification.

In the test, the added excessive soluble CD38 protein bound to daratumumab in patient's serum, the interference thereof was eliminated and the screening and identification of irregular antibodies were successfully restored.^2^ This approach is imperfect in that a large quantity of soluble CD38 receptor is needed against the concentration of the CD38 monoclonal antibody in serum.

It is also plausible to, prior to IAT, eliminate the effects of anti‐CD38 monoclonal antibodies on IAT with specific anti‐CD38 monoclonal antibody neutralizing antibodies ^1^ However, antibodies to anti‐CD38 monoclonal antibodies require high specificity.^13^ The latter two approaches do not interfere with the detection of other like antibodies.

### 3.3 RBC antigen screening prior to treatment

The SIRIUS study reported blood transfusion management and transfusion‐related results in daratumumab‐treated patients. In this study, RBC antigen typing and alloantibody screening were performed in patients prior to treatment. A total of 47 patients received transfusion of 147 units of RBCs and none developed any transfusion reaction or hemolysis, verifying the safety of blood transfusion in daratumumab‐treated patients.^5^ RBC antigen phenotype screening prior to the treatment of anti‐CD38 monoclonal antibodies is the most safe and effective method. It is recommended the assessment cover at least the most common immunogenic antigens, that is, antigens in the ABO, Rh, Duffy, Kidd, Kell, and MNS blood group systems. Previous blood transfusion may affect patients' antigen phenotypes but will not affect antigen genotypes. Genotyping can be performed at any time during treatment and more comprehensive details than phenotyping can be provided, especially with regard to minor antigens.^14^ However, this test is costly and time‐consuming.

## 4 SUMMARY

It is of vital importance to solve problems relating to matching and antibody screening accuracy after the use of anti‐CD38 monoclonal antibodies. Patients should accept RBC antigen phenotype screening prior to transfusion; when making appointments for blood, the blood bank should be notified that the patients have been infused with anti‐CD38 monoclonal antibodies. For patients who have received CD38 monoclonal antibodies, when they need red blood cell transfusion and encounter positive IAT or incompatible crossmatches, a clinical decision should be made according to the patient's condition. Emergency blood transfusion, if needed, should, of course, not be delayed. In emergency, ABO/RhD compatible red blood cells without crossmatching can be used. Rather, the urgency of the clinical condition should be weighed against the risk of allogeneic immunity. If possible, DTT, proteolytic enzymes, and CD38 deficient RBCs from neonatal cord blood could be used to processing of reagent RBC in IAT; papain, excessive soluble CD38 protein, and anti‐CD38 monoclonal antibodies could be used to interference with anti‐CD38 monoclonal antibodies in serum; RBC antigen genotyping could be performed before transfusion. Currently, DTT is the most widely used method. Additionally, it should be noted that positive IAT is not necessarily falsely positive in patients who have received anti‐CD38 monoclonal antibody transfusion; particularly, patients with a history of blood transfusion may develop RBC alloantibodies.

## CONFLICT OF INTEREST

All authors declare no conflict of interest.

## AUTHOR CONTRIBUTIONS

Contributions: J.S. reviewed articles and drafted the manuscript. R.F. ensured the correct and proofed the manuscript. All authors carefully revised the manuscript and finally approved the manuscript.

## References

[1] Chapuy CI , Nicholson RT , Aguad MD , et al. Resolving the daratumumab interference with blood compatibility testing. Transfusion. 2015;55(6 Pt 2):1545‐1554. 25764134 10.1111/trf.13069

[2] Oostendorp M , Jeroen J , van Bueren L , et al. When blood transfusion medicine becomes complicated due to interference by monoclonal antibody therapy. Transfusion. 2015;55(6 Pt 2):1555‐1562. 25988285 10.1111/trf.13150

[3] van de Donk NWCJ , Maarten LJ , Tuna M , et al. Monoclonal antibodies targeting CD38 in hematological malignancies and beyond. Immunol Rev. 2016;270(1):95‐112. 26864107 10.1111/imr.12389 PMC4755228

[4] Palumbo A , Chanan‐Khan A , Weisel K , et al. Daratumumab, bortezomib, and dexamethasone for multiple myeloma. N Engl J Med. 2016;375(8):754‐766. 27557302 10.1056/NEJMoa1606038

[5] Chari A , Arinsburg S , Jagannath S , et al. Blood transfusion management and transfusion‐related outcomes in daratumumab‐treated patients with relapsed or refractory multiple myeloma. Clin Lymphoma Myeloma Leuk. 2018;18(1):44‐51. 29054515 10.1016/j.clml.2017.09.002

[6] Yang X , Xin Y , Ma C , et al. Three cases of cross‐match incompatibility caused by dallimumab. Chin J Blood Transfus. 2020;5(33):529‐531.

[7] Gao X , Wang X , Tian H , et al. Study on the method of blood matching after treatment with Anti‐CD38 monoclonal antibody daratumab in patients with hematologic malignancies. Chin J Blood Transfus. 2020;3(33):227‐230.

[8] Chapuy CI , Aguad MD , Nicholson RT , et al. International validation of a dithiothreitol (DTT)‐based method to resolve the daratumumab interference with blood compatibility testing. Transfusion. 2016;56(12):2964‐2972. 27600566 10.1111/trf.13789

[9] Bub CB . Dithiothreitol treatment of red blood cells. Immunohematology. 2017;33(4):170‐172. 29378150 10.21307/immunohematology-2019-025

[10] Izaguirre EC , Del Mar L‐HM , González LL , et al. New method for overcoming the interference produced by anti‐CD38 monoclonal antibodies in compatibility testing. Blood Transfus. 2020;18(4):290‐294. 32530397 10.2450/2020.0004-20 PMC7375880

[11] Schmidt AE , Kirkley S , Patel N , et al. An alternative method to dithiothreitol treatment for antibody screening in patients receiving daratumumab. Transfusion. 2015;55(9):2292‐2293. 26372917 10.1111/trf.13174

[12] Werle E , Ziebart J , Wasmund E , et al. Daratumumab interference in pretransfusion testing is overcome by addition of daratumumab fab fragments to patients' plasma. Transfus Med Hemother. 2019;46(6):423‐430. 31933572 10.1159/000495773 PMC6944924

[13] McCudden C , Axel AE , Slaets D , et al. Monitoring multiple myeloma patients treated with daratumumab: teasing out monoclonal antibody interference. Clin Chem Lab Med. 2016;54(6):1095‐1104. 27028734 10.1515/cclm-2015-1031

[14] Anani WQ , Marchan MG , Bensing KM , et al. Practical approaches and costs for provisioning safe transfusions during anti‐CD38 therapy. Transfusion. 2017;57(6):1470‐1479. 28150308 10.1111/trf.14021

